# Nitric oxide in exercise physiology: past and present perspectives

**DOI:** 10.3389/fphys.2024.1504978

**Published:** 2025-01-09

**Authors:** Breanna J. Mueller, Michael D. Roberts, Christopher B. Mobley, Robert L. Judd, Andreas N. Kavazis

**Affiliations:** ^1^ School of Kinesiology, Auburn University, Auburn, AL, United States; ^2^ Department of Anatomy, Physiology, and Pharmacology, Auburn University, Auburn, AL, United States

**Keywords:** nitric oxide, vasodilation, skeletal muscle, mitochondria, redox state, nitrate supplementation, exercise performance

## Abstract

Nitric oxide (NO) is a ubiquitous signaling molecule known to modulate various physiological processes, with specific implications in skeletal muscle and broader applications in exercise performance. This review focuses on the modulation of skeletal muscle function, mitochondrial adaptation and function, redox state by NO, and the effect of nitrate supplementation on exercise performance. In skeletal muscle function, NO is believed to increase the maximal shortening velocity and peak power output of muscle fibers. However, its effect on submaximal contraction is still undetermined. In mitochondria, NO may stimulate biogenesis and affect respiratory efficiency. NO also plays a role in the redox state within the skeletal muscle, partially through its interaction with respiratory chain enzymes and transcriptional regulators of antioxidant production. Nitrate supplementation leads to an increased bioavailability of NO in skeletal muscle. Thus, nitrate supplementation has been investigated for its ability to impact performance outcomes in endurance and resistance exercise. The effect of nitrate supplementation on endurance exercise is currently indecisive, although evidence indicates that it may extend the time to exhaustion in endurance exercise. Alternatively, the effect of nitrate supplementation on resistance exercise performance has been less studied. Limited research indicates that nitrate supplementation may improve repetitions to failure. Further research is needed to investigate the influence of training status, age, sex, and duration of supplementation to further elucidate the impact of nitrate supplementation on exercise performance.

## Introduction

Although nitric oxide (NO) has been posited as a deleterious molecule from a physiological perspective (Bryan, 2006), it is also known for its crucial role in maintaining the vascular tone. Furthermore, NO deficiency has been implicated in several pathologies such as cardiovascular diseases (Naseem, 2005), respiratory diseases (Soodaeva et al., 2020), neurodegenerative diseases (Snyder, 1992), and metabolic dysfunction (Litvinova et al., 2015).

In the context of exercise, nitrate supplementation has gained interest as an ergogenic aid to enhance endurance performance (Jones, 2014a) and muscular adaptations to resistance exercise (Gonzalez et al., 2023). Seminal works by Bailey et al. (2009), Jones et al. (2021), Lundberg et al. (2008) have sought to determine how NO affects vascular and muscle physiology and the potential effect of nitrate supplementation on exercise performance . Despite extensive data on this topic, a clear consensus on the effects of nitrate supplementation on exercise performance has yet to be established.

Therefore, the purpose of this review is to present the general knowledge of the underlying physiological mechanisms affected by NO and the subsequent effect of nitrate supplementation in exercise. We begin our review by describing the two pathways that produce NO and summarizing how each pathway can be stimulated to increase production during exercise. Next, we briefly describe the functions of NO in the vasculature (not a focus in this review), followed by a synopsis of the past and present perspectives on NO function within skeletal muscle and the mechanisms therein. We then discuss the effects of NO on mitochondrial biogenesis, respiration, efficiency, and ATP production. The next section expands on NO, mitochondria, and redox state. The final two sections critically appraise the literature, assessing the effects of NO on endurance and resistance exercise performance. We conclude our review by providing proposed future directions on this exciting topic.

### Pathways of NO production

**FIGURE 1:**
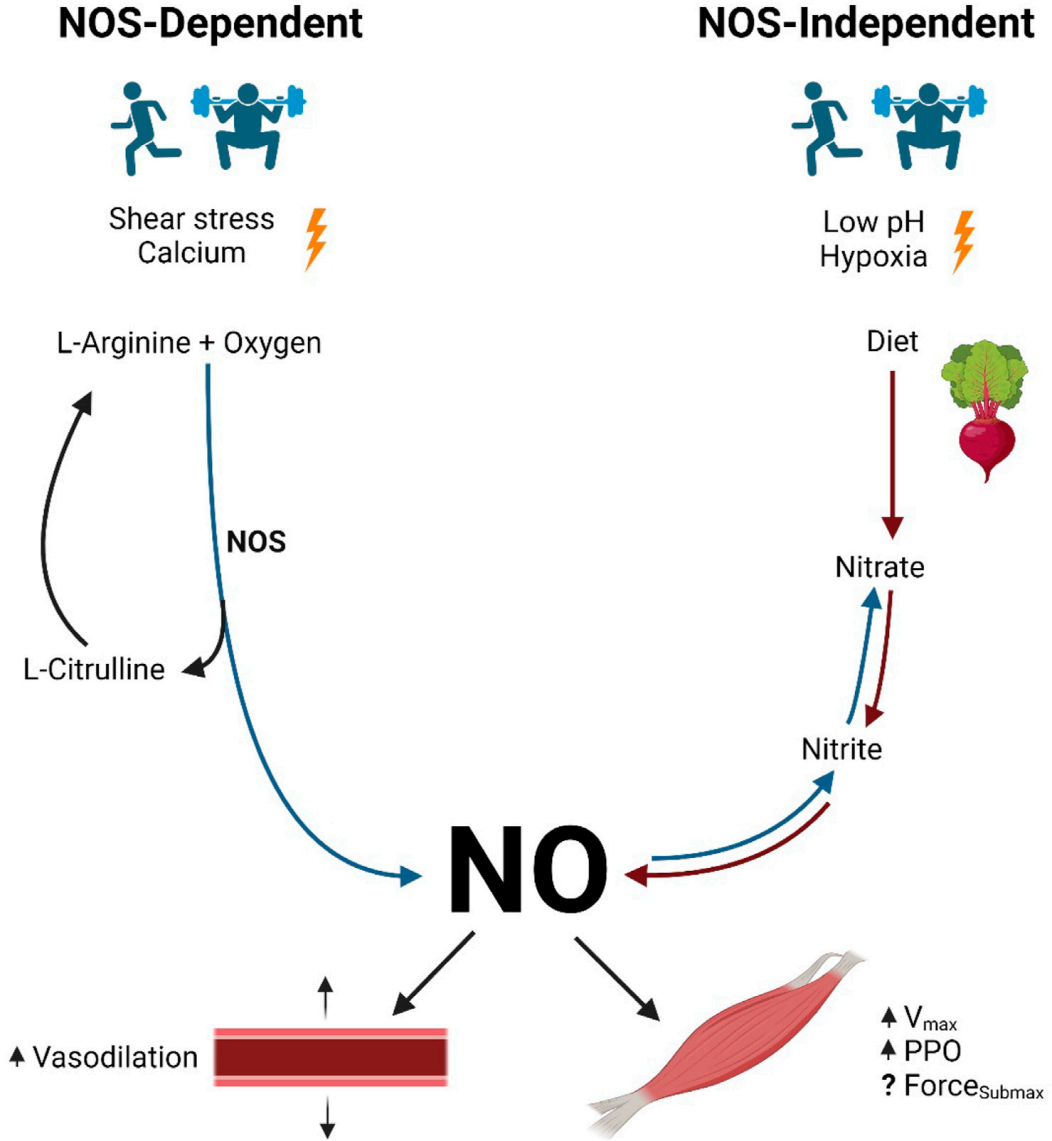
NO production pathways and function. Legend: NOS-dependent pathway: NOS converts L-arginine and oxygen to NO and L-citrulline. NOS-independent pathway: nitrate contained in the diet is reduced to nitrite and then to NO. NO causes vasodilation in arteries. NO increases maximal shortening velocity and peak power output in skeletal muscle, although the results are inconclusive regarding submaximal force production (see text for details). Blue arrows represent oxidation reactions, while red arrows represent reduction reactions. Nitric oxide (NO); nitric oxide synthase (NOS); maximal shortening velocity (V_max_); peak power output (PPO); submaximal force production (Force_Submax_).

NO is a ubiquitous signaling molecule involved with numerous cellular functions, and yet it is restrained by a relatively short half-life. Therefore, it is continuously synthesized throughout the human body (Gilchrist et al., 2011). Certain conditions stimulate an increase in NO production beyond homeostatic concentrations. Depending on the stimulus, NO is produced either with or without nitric oxide synthase (NOS) (i.e., the NOS-dependent and -independent pathways; Figure 1). The following section describes the unique processes involved in the stimulation and production of NO within each pathway.

#### NO production via the NOS-dependent pathway

The NOS-dependent pathway is the primary source of NO in human physiology. In this pathway, NOS catalyzes the conversion of L-arginine and oxygen to L-citrulline and NO (Gonzalez et al., 2023; Ignarro, 1990; Tousoulis et al., 2012). Next, L-citrulline is recycled to re-form L-arginine, while further oxidation of NO yields endogenously produced nitrite and nitrate, some of which are stored for future use and may contribute to NOS-independent NO production (Jones et al., 2018).

NOS-dependent NO production is facilitated by three tissue-specific NOS isozymes, namely, nNOS, eNOS, and iNOS (Förstermann and Sessa, 2012). Since iNOS is primarily activated as an immune and inflammatory response, this review will focus on the action of nNOS and eNOS in NOS-dependent NO production (Förstermann and Sessa, 2012).

#### Stimulation of NO production via the NOS-dependent pathway

nNOS and eNOS are calcium–calmodulin-dependent enzymes responsible for the conversion of L-arginine and oxygen to L-citrulline and NO (Ignarro, 1990; Tousoulis et al., 2012). Therefore, the stimulus necessary to initiate NOS-dependent NO production is an increase in intracellular calcium (Reid, 1998). In endothelial cells, increases in cellular calcium influx via mechanoreceptors and acetylcholine receptors enhance NOS activity (Ignarro, 1990). In skeletal muscle fibers, excitation–contraction coupling leads to the release of calcium from the sarcoplasmic reticulum and increases intracellular calcium concentrations (Stamler and Meissner, 2001). As intracellular calcium concentrations increase, calcium ions bind to calmodulin, forming calcium–calmodulin complexes (Reid, 1998). Finally, calcium–calmodulin complexes activate NOS, which facilitates NOS-dependent NO production (Moncada and Higgs, 1993; Tang et al., 2014).

#### Exercise as a stimulant for NO production via the NOS-dependent pathway

Exercise is a stressor that is known to stimulate NOS-dependent NO production. As exercise begins, cardiac output increases along with blood flow through the vasculature of the working skeletal muscle. As a result, the shear stress imposed on the vasculature walls by these hemodynamic forces is perceived by endothelial cell mechanoreceptors, which facilitates increases in intracellular calcium concentrations. Skeletal muscle contraction also elicits both shear stress to the surrounding arteries due to increased local blood flow and increased muscle fiber intracellular calcium concentrations via excitation–contraction coupling (Stamler and Meissner, 2001). As a result of shear stress on the vasculature and skeletal muscle contractions during exercise, endothelial and myocellular calcium concentrations increase, leading to the activation of NOS in these cell types and enhanced NO production (Kolluru et al., 2014).

#### NO production via the NOS-independent pathway

In the NOS-independent pathway, NO production is the result of the sequential reduction of nitrate to nitrite to NO. Although small amounts of nitrite formed as an end product of the NOS-dependent pathway contribute to the nitrate–nitrite–NO pathway, the most abundant source of nitrite-derived NO is the reduction of nitrates consumed in the diet. Upon entering the mouth, approximately 20% of dietary nitrate is immediately converted to nitrite by commensal facultative anaerobic bacteria found under the tongue (Duncan et al., 1995; Gilchrist et al., 2010; Liu G. et al., 2023; Lundberg and Weitzberg, 2010). Next, the acidic environment of the stomach facilitates further reduction of any swallowed salivary nitrite into NO (Benjamin et al., 1994; Lundberg and Weitzberg, 2010). The remaining ingested nitrite is stored in the tissues for future reduction into NO (Jones et al., 2021).

#### Stimulation of NO production via the NOS-independent pathway

Although several investigations have provided insight into the impact of the NOS-independent pathway, research has yet to reveal the specific stimuli required to initiate this pathway (Lundberg et al., 2008). However, there are two conditions under which the NOS-independent pathway is known to be enhanced: low oxygen (hypoxia) (Cosby et al., 2003; Zweier et al., 1995) and low pH (acidosis) (Jones et al., 2021; Zweier et al., 1999).

#### Exercise as a stimulant for NO production via the NOS-independent pathway

The two conditions mentioned above (hypoxia and/or acidosis) commonly occur with high-intensity and intermittent exercise (Jones et al., 2021). During intense exercise, when greater demands are placed on the working skeletal muscle, oxygen use drastically increases via mitochondrial respiration, thereby inducing local hypoxia. Furthermore, skeletal muscle also requires energy (i.e., ATP) to be rapidly produced during high-intensity bouts, thereby drawing on lactic acid-producing anaerobic energy systems. This idea is supported by the results of Piknova et al. (2016), who demonstrated a decrease in skeletal muscle nitrate concentration and an increase in nitrite concentration following a bout of exercise. Therefore, exercise may be a potent stimulus for NOS-independent NO production.

### Functions of NO in the vasculature

Once synthesized, NO is involved in several physiological functions. The effect of NO on the vasculature is well-documented and involves the diffusion of NO from the endothelial cell, where it is synthesized, into the neighboring smooth muscle cells. This facilitates the relaxation of the vascular smooth muscle via either the cyclic guanosine monophosphate (cGMP)-dependent pathway or the cGMP-independent pathway (Tiidus et al., 2012). In the cGMP-dependent pathway, NO activates an enzyme called soluble guanylate cyclase (GC) (Tiidus et al., 2012). GC promptly catalyzes the conversion of guanosine triphosphate (GTP) into cGMP (Moncada and Higgs, 1993; Tiidus et al., 2012). Increasing concentrations of cGMP within the smooth muscle cells activate protein kinase G, which ultimately reduces intercellular calcium concentrations, relaxes smooth muscle cells, and dilates associated blood vessels (Lincoln et al., 2001). In the cGMP-independent pathway, NO alternatively activates the sarco/endoplasmic reticulum calcium ATPase pump. The sarco/endoplasmic reticulum calcium ATPase pump transports intracellular calcium into the sarcoplasmic or endoplasmic reticulum of the cardiac, skeletal, and vascular smooth muscle cells. Resultant reductions in intracellular calcium concentrations lead to smooth muscle relaxation and vasodilation (Adachi et al., 2004). Since this topic is not the focus of this review, readers interested in more details are directed to other excellent reviews on the topic (Cyr et al., 2020; Jin and Loscalzo, 2010; Maiorana et al., 2003).

### Functions of NO in skeletal muscle

**FIGURE 2:**
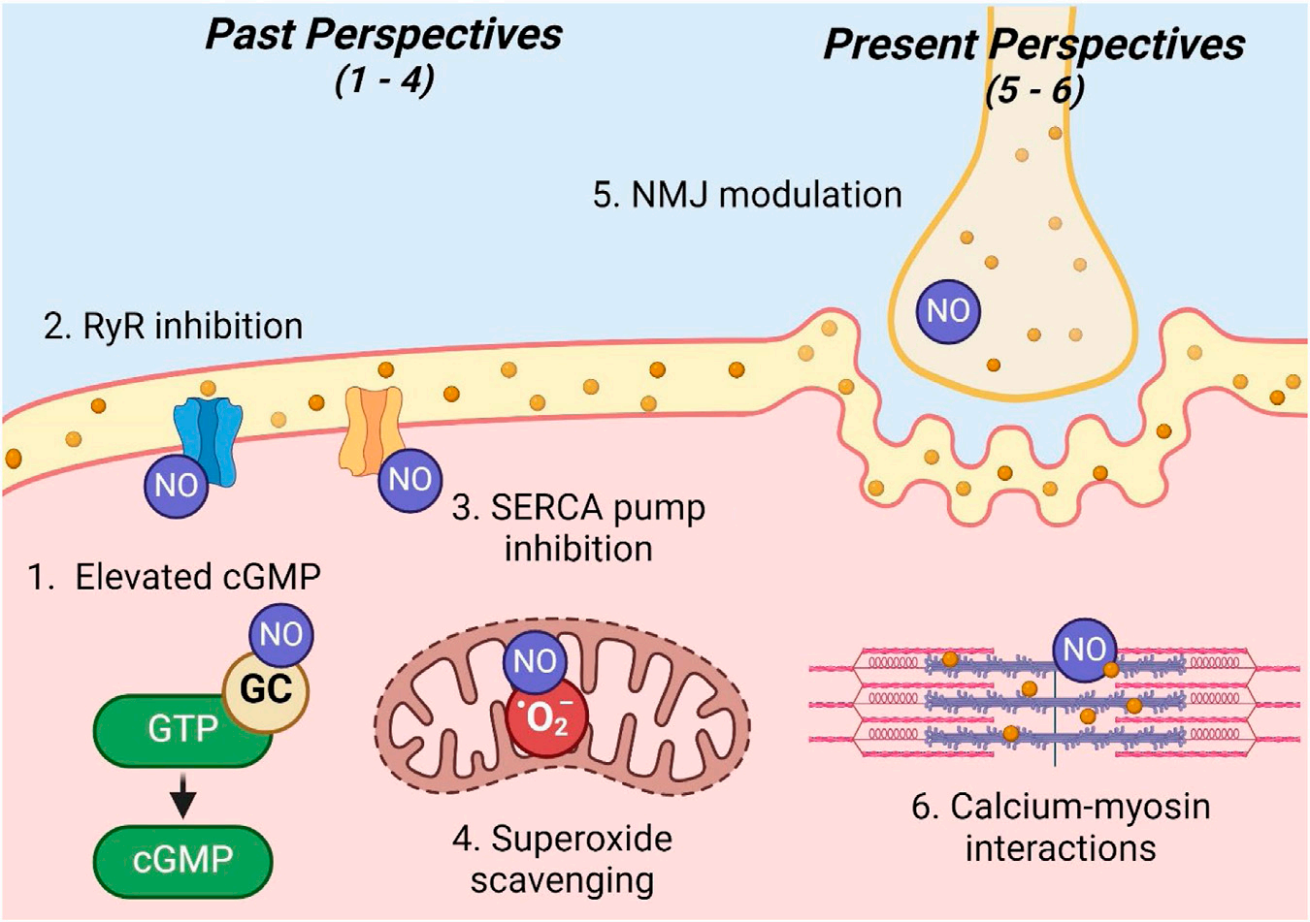
Past and present perspectives on NO skeletal muscle modulation. Legend: sites 1–4 illustrate past perspectives, while sites 5 and 6 illustrate present perspectives of the modulation of skeletal muscle contraction by NO: 1) elevated cGMP; 2) ryanodine receptor inhibition; 3) sarco/endoplasmic reticulum calcium ATPase pump inhibition; 4) superoxide scavenging; 5) neuromuscular junction modulation; 6) calcium–myosin interactions. See text for details. Nitric oxide (NO); ryanodine receptor (RyR); guanylate cyclase (GC); guanosine triphosphate (GTP); cyclic guanosine monophosphate (cGMP); sarco/endoplasmic reticulum calcium ATPase (SERCA); superoxide (O_2_^−^); neuromuscular junction (NMJ).

Since the discovery of its presence in skeletal muscle (1994), NO has been studied for its effects on contractile function (Kumar et al., 2022). However, some effects are still undetermined (Kumar et al., 2022). How NO affects muscle contractile function is likely dependent on factors such as the NO production pathway, intracellular concentrations of NO/NO donors, dietary nitrate intake, the environment in which the skeletal muscle model is studied (*in vivo* vs. *ex vivo*), the muscle preparation (isolated or intact), the primary fiber type utilized, and other factors (Kumar et al., 2022; Stamler and Meissner, 2001). Conflicting results have made it apparent that the effect of NO on skeletal muscle contractile function is influenced by a complex array of conditions. Although several studies collectively suggest that NO enhances the maximal shortening velocity and peak power output in skeletal muscle, past and present investigations have produced opposing perspectives on the effect of NO on submaximal force production (Kumar et al., 2022). The following sections summarize the past and present perspectives on the underlying mechanisms by which NO modulates skeletal muscle function (Figure 2).

#### Past perspectives

Previous reports by several authors, such as Maréchal and Gailly (1999); Morrison et al. (1996), Reid (1998), Stamler and Meissner (2001), agree with the present perspective that NO functions to increase the maximal shortening velocity and peak power output; however, these studies also suggest a decrease in submaximal force production in skeletal muscle that is contrary to the present perspective. NO was proposed to modulate contractile function via the following four mechanisms within skeletal muscle.

First, similar to endothelial cells, NO acts on GC to increase the levels of cGMP in skeletal muscle. Although the actual mechanism of cGMP interactions was not specified, authors postulated that cGMP in skeletal muscle likely operates in a manner similar to its mechanism of muscle relaxation in smooth muscle. Ultimately, several studies conducted in the 1990s demonstrated a decrease in submaximal force production (Abraham et al., 1998; Kobzik et al., 1994) and an increase in the maximal shortening velocity (Maréchal and Beckers-Bleukx, 1998) in direct response to increased concentrations of cGMP.

Second, NO was proposed to decrease submaximal force production by influencing calcium release. Specifically, it was theorized that NO inhibits ryanodine receptors, thereby decreasing calcium release and subsequent cross-bridge formation (Reid, 1998; Stamler and Meissner, 2001). This inhibition was proposed to occur either through a direct interaction of NO with ryanodine receptors to decrease their function or by NO scavenging reactive oxygen species, which enhances ryanodine receptor calcium release (Kobzik et al., 1994; Mészáros et al., 1996). However, it is important to note that some other studies at this time observed a contradictory response, where increased NO resulted in the activation of ryanodine receptors calcium release channels (Aghdasi et al., 1997; Stoyanovsky et al., 1997).

Third, NO may modulate contractile function via the regulation of calcium uptake. The NO-mediated inhibition of sarco/endoplasmic reticulum calcium ATPase pumps was believed to facilitate sustained calcium release, thereby increasing cytosolic calcium concentrations and cross-bridge formation (Stamler and Meissner, 2001; Xu et al., 1999).

Finally, NO may modulate contractile function via its role as an antioxidant. Research has shown that superoxide, a reactive oxygen species, optimizes force production in skeletal muscle (Reid et al., 1993). Typically, reactive oxygen species concentrations are balanced by the antioxidant superoxide dismutase. However, NO can scavenge superoxide at a rate three times more effective than superoxide dismutase (Stamler and Meissner, 2001). Ultimately, this effect of NO may negatively impact contractile function by countering the positive action of reactive oxygen species to optimize contractile function.

#### Present perspectives

Although recent publications from authors such as Kumar et al. (2022) confirm previous claims that NO functions to increase the maximal shortening velocity and peak power output in skeletal muscle, they do not agree on the effect of NO on submaximal force production. Rather, these authors postulate that NO functions to increase submaximal force production. These authors also propose significantly different mechanisms for modulating contractile function. Although the previous school of thought centers around cGMP pathways, calcium handling, and antioxidant capacity, new evidence illuminates excitation–contraction coupling and myofilament function as primary mechanisms modulating contractile function.

First, NO may affect contractile function via its involvement at the neuromuscular junction (Kumar et al., 2022). Specifically, recent evidence suggests that NO signaling triggers vesicle release (Wildemann and Bicker, 1999), enhances neurotransmitter release (Nickels et al., 2007), and inhibits acetylcholinesterase activity (Petrov et al., 2013). Enhancement of any one of these actions may serve to improve neuromuscular transmission, leading to enhanced skeletal muscle contraction.

It is well-established that NO regulates intracellular calcium via ryanodine receptor activation and sarco/endoplasmic reticulum calcium ATPase pump inhibition (Kumar et al., 2022; Reid, 1998; Stamler and Meissner, 2001). Originally, the mechanism of modulation was attributed to subsequent increased calcium-actin binding and increased cross-bridge formation (Kumar et al., 2022). However, since calcium-actin binding naturally occurs at a rate much faster than normal force development, it is unlikely to be a limiting factor and, thus, a mechanism of enhanced contractile function. Rather, calcium–myosin interactions are much slower and, consequently, serve as a more likely mechanism for the modulation of contractile function (Kumar et al., 2022). Therefore, the authors suggest that contractile function is enhanced via NO acting to quicken the rate of cross-bridge cycling (cross-bridge kinetics) (Kumar et al., 2022). Finally, it is important to note that enhanced muscle contraction may be due to one or both of the mechanisms described above (Kumar et al., 2022).

### NO and mitochondria

#### Role of NO in mitochondrial biogenesis

**FIGURE 3:**
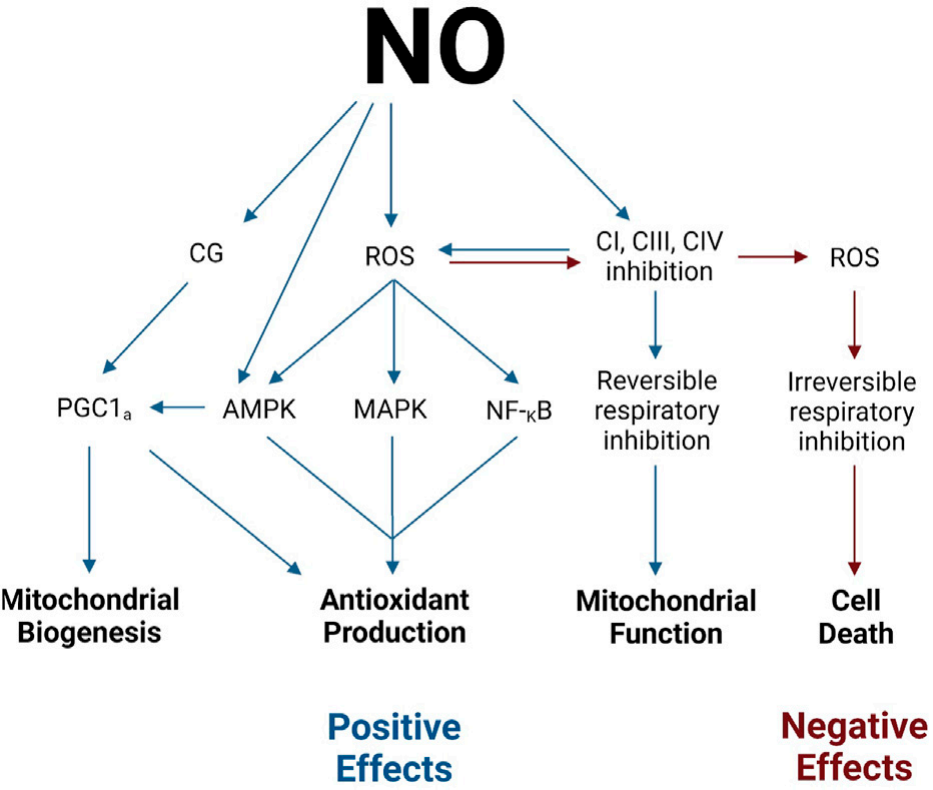
Positive and negative effects of NO. Legend: NO stimulates mitochondrial biogenesis through the activation of PGC-1α via CG and AMPK pathways. NO stimulates antioxidant production via PGC-1α, AMPK, MAPK, and NF-κB activation. NO may modulate mitochondrial function via the direct reversible inhibition of electron transport chain complexes. NO may stimulate cell death via the overproduction of ROS and subsequent irreversible respiratory inhibition (see text for details). See text for details. Blue arrows indicate positive effects, while red arrows indicate negative effects. Nitric oxide (NO); guanylate cyclase (GC); reactive oxygen species (ROS); proliferator-activated receptor gamma coactivator-1 alpha (PGC-1α); 5′-AMP-activated protein kinase (AMPK); p38 mitogen-activated protein kinase (MAPK); nuclear factor-κB (NF-κB); complex I (CI); complex III (CIII); complex (IV).

Although the underlying mechanisms are not fully understood, several studies indicate an interaction between NO and a mitochondrial biogenesis regulator peroxisome proliferator-activated receptor gamma coactivator-1 alpha (PGC-1α) (Lira et al., 2010; Nisoli and Carruba, 2006; Tengan et al., 2007; 2012) (Figure 3). NO is believed to increase PGC-1α activity via the activation of the GC pathway or the 5′-AMP-activated protein kinase pathway (Tengan et al., 2012).

As previously discussed, NO activates GC and the subsequent production of cGMP, which modulates muscle contractile function. However, increased cGMP concentration has also been observed to coincide with (NO-mediated) increases in mitochondrial biogenesis (Nisoli et al., 2003). Once activated by NO, cGMP initiates a slew of phosphorylation events. First, cGMP activates serine/threonine protein kinase A. Protein kinase A, in turn, activates cAMP responsive element-binding protein 1, which translocates from the cytosol into the nucleus (Tengan et al., 2012). cAMP-responsive element-binding protein 1 is a potent activator of PGC-1α and, thereby, initiates the synthesis of mitochondrial and respiratory chain proteins (Tengan et al., 2012; Wu et al., 2006). Ultimately, the activation of GC by NO leads to the stimulation of PGC-1α and NO-mediated mitochondrial biogenesis.

Alternatively, NO also stimulates mitochondrial biogenesis via the activation of 5′-AMP-activated protein kinase (AMPK) (Tengan et al., 2012). This heterotrimeric kinase is well-known as an activator of PGC-1α (Reznick and Shulman, 2006). A recent study by McConell et al. (2010) confirmed the relationship between AMPK, PGC-1α, and NO in L6 myogenic cells. Therefore, as NO concentrations increase, subsequent stimulation of AMPK results in PGC-1α activation and NO-mediated mitochondrial biogenesis.

#### Role of NO in mitochondrial function

Nitric oxide regulates mitochondrial function both indirectly by modulating systemic responses and directly by interacting with the electron transport chain complexes. As previously mentioned, in the cardiovascular system, NO dilates the blood vessels of working skeletal muscle. The resulting increase in blood flow to working skeletal muscles provides a greater supply of respiratory substrates and oxygen to the mitochondria (Tengan et al., 2012). Thus, the role of NO in the cardiovascular system indirectly supports mitochondrial function.

**FIGURE 4:**
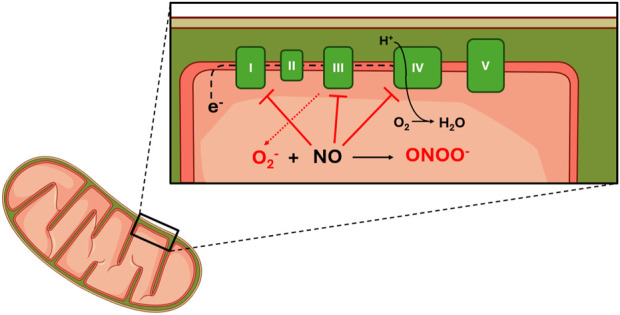
Effect of NO on mitochondrial function. Legend: NO blocks electron flow through complexes I, III and IV. Binding of NO at CIII results in increased superoxide production. No binds at CIV competitively with oxygen. Superoxide reacts with No to increase peroxinitrite production. See text for more details. Complex I (I); Complex II (II); Complex III (III); Complex IV (IV); Complex V (V); Electrons (e^−^); Superoxide (O_2_^−^); Hydrogen (H^+^); Dihydrogen monoxide (H_2_O).

Within the mitochondria itself, NO directly impacts mitochondrial respiration and redox state by interacting with the individual complexes of the electron transport chain (Figure 4) (Tengan et al., 2012). Specifically, NO functions to inhibit electron transfer (decreasing activity) at complexes I (CI), III (CIII), and IV (CIV) and generates superoxide anions at CIII (Boveris et al., 2000; Taylor and Moncada, 2010; Tengan et al., 2012). At CI, NO functions to obstruct electron transfer by mechanisms that have not yet been fully elucidated (Tengan et al., 2012). However, research indicates that S-nitrosation, tyrosine nitration, and damage to Fe–S centers may play a role (Boveris et al., 2000; Pappas et al., 2023; Tengan et al., 2012).

At CIII, studies have observed decreased electron transfer and increased free radical generation following increased intracellular NO concentrations (Moncada and Erusalimsky, 2002; Poderoso et al., 1996). The effects of increased free radical generation at CIII on the redox state will be discussed in greater detail later in this review. Although earlier research suggested that NO imposes its effect via direct interaction with the cytochrome bc1 complex (submitochondrial particles found within CIII) (Poderoso et al., 1996; 2019), recent research presents three alternative hypotheses. Summarizing past and present hypotheses, decreased electron flow through CIII may be the result of 1) a direct effect of NO (original hypothesis), 2) an indirect effect of the mechanism responsible for NO production (S-nitrosoglutathione/dithiothreitol system), 3) increased formation of peroxynitrite from NO and superoxide, or 4) an effect of NO on the electron transport carrier ubiquinol-10 (Poderoso et al., 2019).

**FIGURE 5:**
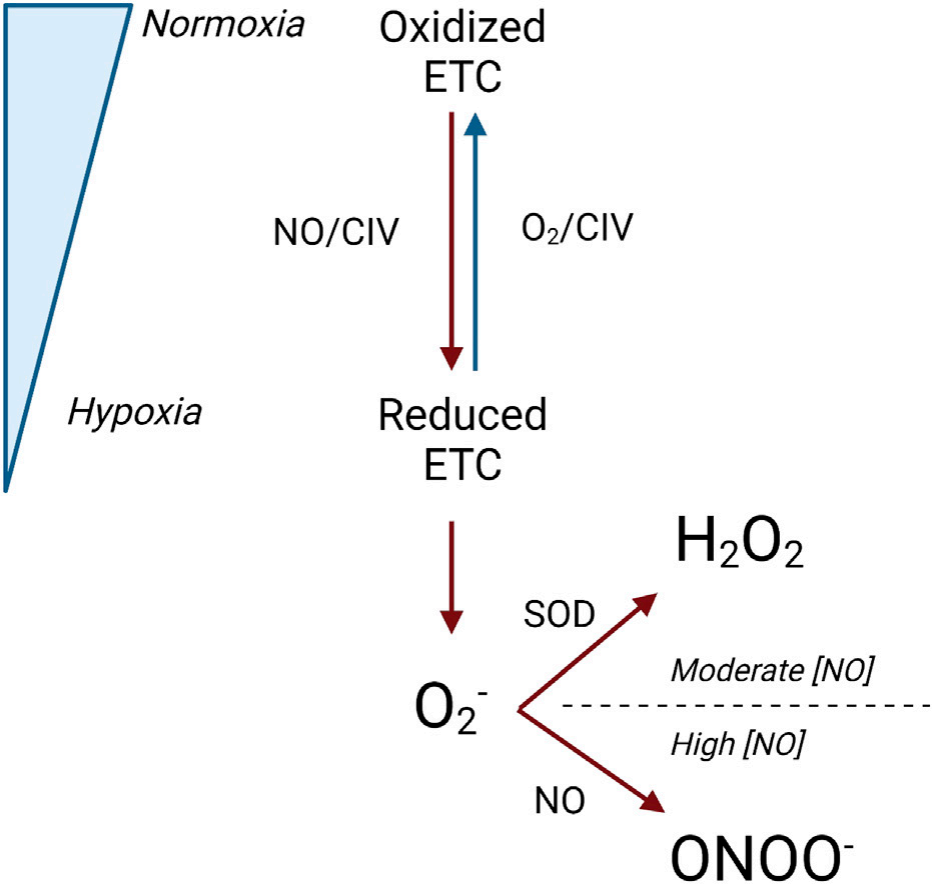
Interaction between the redox state of complex IV and NO. Legend: Under normoxic conditions, the electron transport chain is primarily oxidized, and oxygen and NO can concurrently bind at complex IV. Under hypoxic conditions, the electron transport chain is primarily reduced, and NO competes with oxygen to bind at complex IV. When this occurs, superoxide is produced and can be converted to either hydrogen peroxide by superoxide dismutase or peroxynitrite by NO. Blue arrows represent oxidation reactions, while red arrows represent reduction reactions. See text for details. Nitric oxide (NO); electron transport chain (ETC); complex IV (CIV); oxygen (O_2_); superoxide (O_2_^−^); superoxide dismutase (SOD); peroxynitrite (ONOO^−^); nitric oxide concentration ([NO]).

Although inhibited electron transfer at CI and CIII contributes to inhibited mitochondrial respiration, the action of NO at CIV may be the primary means of respiratory inhibition within the mitochondria. At CIV, NO inhibits respiration by binding to its oxygen binding site and inhibiting the flow of electrons through the respiratory chain (decreasing complex activity) (Taylor and Moncada, 2010; Tengan et al., 2012). However, both the mechanism and degree of respiratory inhibition are dependent on intracellular oxygen concentration and the redox state of CIV (Figure 5) (Taylor and Moncada, 2010).

NO binds to CIV at both normoxic oxygen concentrations, where CIV is primarily oxidized, and hypoxic oxygen concentrations, where CIV is primarily reduced. However, the specific conditions under which NO binds with the oxygen-binding site on CIV may or may not concurrently cause the obstruction of oxygen binding. The oxygen-binding site on CIV is comprised of a binuclear heme iron/copper center, which exists in an oxidized or reduced state depending on the surrounding oxygen concentrations (Pappas et al., 2023). When CIV is predominantly oxidized at normoxic oxygen concentrations, NO binds to the copper center in a manner that does not obstruct concurrent oxygen binding (Brown and Borutaite, 2007; Pappas et al., 2023; Taylor and Moncada, 2010). Thus, respiratory function and oxygen consumption are maintained (Pappas et al., 2023; Taylor and Moncada, 2010). Under these conditions, NO also reacts with oxygen to form nitrite (Brown and Borutaite, 2007). Additionally, normal respiratory activity and oxygen consumption are also aided through the recruitment of previously inactive CIV enzymes, which can maintain electron transfer and oxygen consumption (Chance, 1988; Taylor and Moncada, 2010). When CIV is predominantly reduced at hypoxic oxygen concentrations, NO binds to the iron center of the oxygen-binding site, and oxygen must compete with NO as the final electron acceptor (Brown and Borutaite, 2007; Cooper and Giulivi, 2007; Pappas et al., 2023). Because CIV has a greater affinity for NO than oxygen, NO binds to CIV instead of oxygen. As a result, respiration is inhibited, and oxygen consumption is reduced (Taylor and Moncada, 2010; Tengan et al., 2012).

Although it has been established that NO decreases the flow of electrons through the respiratory chain complexes, which inhibits respiration and decreases oxygen consumption (Giulivi et al., 2006), the physiological impact of this effect has yet to be fully elucidated. There are two possible outcomes of NO-mediated respiratory inhibition. Some authors infer a null or negative effect of NO on mitochondrial function and total energy production, although specific mechanisms have yet to be determined (Ntessalen et al., 2020; Whitfield et al., 2016). For example, one study concluded that despite a decrease in submaximal oxygen consumption, mitochondrial efficiency and coupling were not affected (Whitfield et al., 2016). Furthermore, these authors reported adverse effects of an increased rate of mitochondrial hydrogen peroxide production (Ntessalen et al., 2020). However, others propose that respiratory inhibition negatively affects ATP production via subsequent effects on mitochondrial depolarization (Balakirev et al., 1997; Brown and Borutaite, 2007).

Alternatively, there is the notion that NO advantageously affects mitochondrial efficiency (Pappas et al., 2023). Specifically, efficiency is improved when NO acts as the final electron acceptor and simultaneously maintains ATP production while sparing oxygen consumption (Cooper and Giulivi, 2007). This was demonstrated by Larsen et al. (2007), where submaximal oxygen cost was decreased and work output was maintained (Larsen et al., 2007). These outcomes were attributed to increased respiratory efficiency since these results were not accompanied by increased lactate concentration, which would have indicated a compensatory increase in anaerobic energy production (Larsen et al., 2007). From this standpoint, the ultimate effect of NO-mediated respiratory inhibition is improved mitochondrial efficiency (Boveris et al., 2000; Giulivi, 2003; Tengan et al., 2012).

### NO and redox state

Not only does NO regulate mitochondrial function and biogenesis via interactions with signaling molecules and the electron transport chain, but it also regulates the production of mitochondrial free radicals (reactive oxygen species and reactive nitrogen species) (Moncada and Erusalimsky, 2002; Pappas et al., 2023) (Figure 3). As previously mentioned, the interaction of NO with CI and CIII inhibits electron flow through the respiratory complexes (Tengan et al., 2012). As a result, the respiratory complexes shift toward a more reduced state, which augments superoxide production (Boveris and Chance, 1973; Moncada and Erusalimsky, 2002; Tengan et al., 2012). Superoxide, in turn, can react with either superoxide dismutase to form hydrogen peroxide or with NO to form peroxynitrite (Moncada and Erusalimsky, 2002). The production of hydrogen peroxide or peroxynitrite from superoxide is determined by the concentration of NO present. Moderate concentrations of NO can increase superoxide and hydrogen peroxide production, while higher concentrations of NO scavenge superoxide to inhibit hydrogen peroxide production and increase peroxynitrite production (Riobó et al., 2001). The downstream effects of increased NO-induced production of free radicals can have both positive and negative ramifications.

#### Positive effects—Antioxidant production

A potential positive effect of NO on the redox state is that NO can stimulate antioxidant production pathways (Ji et al., 2007; Mason et al., 2016). NO is linked to antioxidant production through its involvement in free radical generation and PGC-1α activation. It is well-established that oxidative stress concurrently signals counteractive antioxidant production pathways (Jomova et al., 2023). Specifically, increased reactive oxygen species is known to activate the AMPK, p38 mitogen-activated protein kinase, and nuclear factor-κB pathways, each of which plays a role in antioxidant production pathways (Irrcher et al., 2009; Mason et al., 2016). Therefore, NO inadvertently activates antioxidant defense systems through reactive oxygen species signaling. Additionally, as previously mentioned, NO stimulates the expression of PGC-1α. Among many other signaling pathways, PGC-1α also stimulates downstream antioxidant production (Mason et al., 2016).

Moreover, although NO itself is a reactive nitrogen species, it can also serve as an antioxidant. As previously mentioned, at high concentrations, NO tends to combine with superoxide to form peroxynitrite (Wink et al., 1999; 2001). Peroxynitrite is a potent form of ROS, which can have deleterious effects on neighboring molecules. However, upon the formation of peroxynitrite from NO and superoxide, peroxynitrite immediately rearranges to form nitrate when other ROS are not present (Wink et al., 1999; 2001). Thus, NO ultimately acts as an antioxidant by shunting superoxide to peroxynitrite to nitrate.

#### Negative effects—inhibit mitochondrial respiration and/or induce cell death

Although the exact mechanisms are undetermined, peroxynitrite is known to irreversibly inhibit mitochondrial respiration. Peroxynitrite is believed to inhibit electron flow at CI, CIII, and minimally at CIV via direct interaction with the complexes (Poderoso et al., 2019). However, other mechanisms proposed include the modification of protein thiols, destruction of iron–sulfur centers contained in CI and CII, or opening of the permeability transition pore, which results in the loss of cytochrome c and contributes to respiratory inhibition (Imaizumi and Aniya, 2011; Poderoso et al., 2019; Radi et al., 2002).

Furthermore, increased free radical production can also induce cell death via the downstream effects of oxidative damage to the respiratory chain and RNS-mediated induction of diminished mitochondrial permeability transition (Poderoso et al., 2019). Although a moderate degree of respiratory inhibition may only affect respiratory efficiency, more severe respiratory inhibition may result in cell death. Such respiratory inhibition can result from the additive effects of NO and the oxidative damage on the respiratory complexes. While the previously described NO-mediated respiratory inhibition is reversible, free radical-mediated respiratory inhibition is irreversible. Additionally, respiratory inhibition itself further increases free radical production. Ultimately, the compounding effect of NO and oxidative damage within the mitochondria can spiral into a vicious cycle, where increasing respiratory inhibition produces more free radicals, which further increases respiratory inhibition and induces cell death (Poderoso et al., 2019; Buetler et al., 2004).

Alternatively, RNS-mediated induction of mitochondrial permeability transition may also signal (necrotic or apoptotic) cell death (Poderoso et al., 2019). Mitochondrial permeability transition is a distinct increase in the permeability of the inner mitochondrial membrane, which can diminish the protonmotive force and depolarize the membrane. The result is the mitochondrial uncoupling of oxidative phosphorylation and reversal of ATP synthase. The combined impact of both a decreased ability to produce ATP and increased ATP consumption ultimately triggers (necrotic) cell death. Additionally, mitochondrial permeability transition may cause the outer mitochondrial membrane to swell, burst, and release cytochrome c and mitochondrial matrix components such as apoptogenic factors into the cytosol, which may signal (apoptotic) cell death (Poderoso et al., 2019).

There is no doubt that NO plays a key role in the redox state; however, the positive or negative effect of NO’s role in enhancing the production of reactive oxygen species ultimately depends on the balance between reactive oxygen species and antioxidant production (Pappas et al., 2023).

### Effect of nitrate supplementation on endurance exercise performance

Considering the impact of NO on blood flow regulation and its putative signaling roles in skeletal muscle, mitochondria, and redox state, it is not surprising that exercise physiologists have sought to apply its value to exercise performance. The NOS-independent pathway in skeletal muscle and subsequent nitrate concentration can be enhanced through dietary nitrate supplementation. Dietary nitrate is attained from several food sources, such as spinach and beetroot juice (Jones et al., 2021; Piknova et al., 2016) and can be stored in skeletal muscle for later use (Gilliard et al., 2018). Once ingested, transport from the bloodstream into the skeletal muscle is primarily facilitated by anion transporters, although some nitrate enters skeletal muscle via diffusion (Jones et al., 2021). Skeletal muscle stores nitrate for both its own use and as a reservoir for other organs (Jones et al., 2021; Piknova et al., 2016). The primary role of the NOS-independent pathway in skeletal muscle was demonstrated in a study by Piknova et al., where xanthine oxidoreductase inhibition (an enzyme that is essential to the NOS-independent pathway) inhibited NO production in skeletal muscle; however, NOS inhibition had no effect (Jones et al., 2021; Piknova et al., 2016). Additionally, the reduction of nitrate to nitrite and then to NO in the NOS-independent pathway is enhanced at low pH (6.5), a condition commonly encountered within active skeletal muscle (Jones et al., 2021; Piknova et al., 2016). The final piece of evidence in favor of the activation of the NOS-independent pathway in skeletal muscles during exercise is the observation that nitrate stores are depleted while nitrite concentrations are elevated post skeletal muscle contraction (Piknova et al., 2016).

**FIGURE 6:**
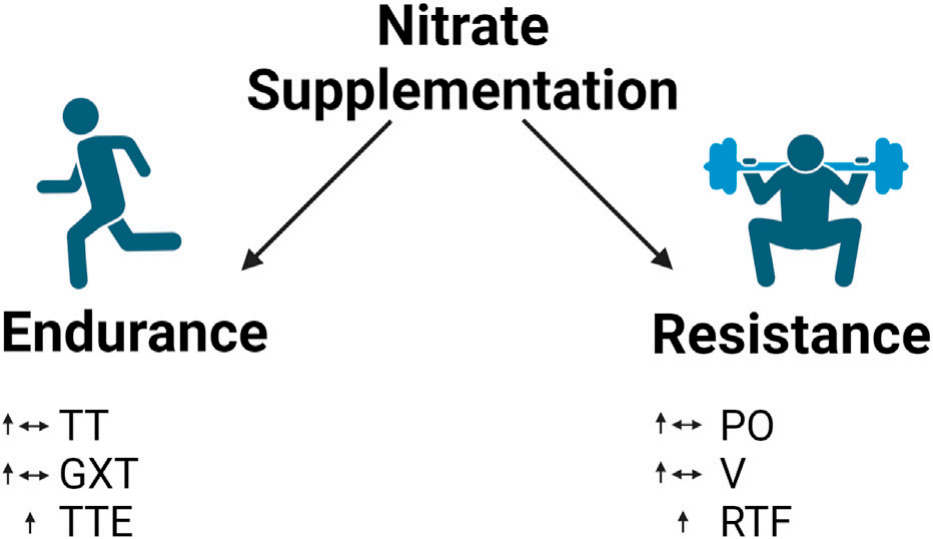
Effect of nitrate supplementation in endurance and resistance exercise. Legend: endurance exercise: mixed results in time trials and graded exercise tests but increase in time to exhaustion. Resistance exercise: mixed results in power output and contraction velocity but increase repetitions to failure. See text for details. Time trial (TT); graded exercise test (GXT); time to exhaustion (TTE); power output (PO); contraction velocity (V); repetitions to failure (RTF).

Meta-analyses by Hoon et al. (2013) and McMahon et al. (2017) surveyed the effects of nitrate supplementation on exercise tolerance and endurance performance. In these studies, graded exercise tests and time-to-exhaustion tests assessed exercise tolerance, while time trials measured endurance exercise performance (Van De Walle and Vukovich, 2018). Both studies suggest that nitrate supplementation elicited small-to-moderate improvements in time trials, graded exercise tests, and time to exhaustion; however, only time to exhaustion reached statistical significance (Figure 6).

#### Exercise tolerance—graded exercise tests and time to exhaustion

A recent meta-analysis detected only a small trend toward improvement in graded exercise test performance, and a subgroup analysis revealed a statistically significant improvement in time to exhaustion following nitrate supplementation. Lansley et al. and Bailey et al. report key findings on graded exercise tests and time to exhaustion following nitrate supplementation. Lansley et al. recruited nine healthy, physically active male candidates to complete graded exercise tests following six days of either placebo or nitrate supplementation in a randomized, crossover experimental design. Nitrate supplementation resulted in reduced oxygen uptake (VO_2_) at moderate and severe intensities and a reduced VO_2_ at the time of exhaustion (Lansley et al., 2011b). Likewise, Bailey et al. conducted a study assessing the effect of nitrate supplementation on graded exercise test performance and time to exhaustion. This investigation also implemented a randomized, crossover experimental design, where seven recreationally trained male candidates completed a series of graded exercise tests at high (115 rpm) and low (35 rpm) intensities on a cycle ergometer. Either placebo or nitrate supplementation was consumed beforehand. Although the results reported by Bailey et al. (2015) did not reveal a statistically significant effect on VO_2_ at the time of exhaustion, there was a statistically significant increase in time to exhaustion. Although the literature does not agree on the exact effect of nitrate supplementation on all measures of exercise tolerance, scientists agree that further investigation is needed.

#### Endurance exercise performance—time trials

Lansley et al. (2011a) and Cermak et al. (2012b) demonstrated improved time trial performance with both acute and chronic nitrate supplementation, respectively. This effect is attributed to improvements in exercise economy and power output. Exercise economy refers to a reduced oxygen cost at a given work rate, and the power output is the product of force production and contraction velocity. Mechanisms responsible for these outcomes are likely due to the effect of NO on skeletal muscle and mitochondria, as discussed in previous sections (Larsen et al., 2012). In the study by Lansley et al., nine competitive cyclists consumed either a placebo or nitrate supplement 2.5 h before completing a 4- and 16.1-km cycling time trial in a random, crossover design. This study demonstrated an increase in cycling economy since nitrate supplementation simultaneously increased the mean power output without increasing the oxygen cost (i.e., oxygen uptake (VO_2_) remained the same). As a result, cyclists had improved time trial performance at both distances following acute nitrate supplementation. Cermak et al. demonstrated improved time trial performance via enhanced exercise economy following chronic nitrate supplementation (Cermak et al., 2012). Before and after six days of nitrate supplementation, 12 trained cyclists completed three exercise tests, including a submaximal exercise bout at both 45% and 65% of peak oxygen uptake (VO_2_ peak), followed by a 10-k time trial. Once again, improved time trial performance was accompanied by increased mean peak power output. Submaximal VO_2_ was lower during exercise bouts at 45% and 65% VO_2_ peak. Collectively, these data indicate that both acute and chronic nitrate supplementation is an effective means of improving exercise economy via an increase in peak power output and a concurrent reduction in submaximal VO_2_.

Although these results provide a compelling case for nitrate supplementation to improve aspects of endurance performance, it is important to note that there is not unanimous agreement on these conclusions. Several studies implementing similar study designs yielded non-significant findings (Cermak et al., 2012a; Jones, 2014b; Wilkerson et al., 2012). Although other limitations may be present, one may note that conflicting results between similar study designs may be due to limitations within the study itself, such as underpowered sample sizes and discrepancies in supplementation protocols (Abt et al., 2020; Houtvast et al., 2024). Furthermore, many studies employ varying study designs and methodologies, which may contribute to incongruent findings across nitrate supplementation research. For example, studies investigating graded exercise tests, time to exhaustion, and time trial performance often differ in nitrate supplementation (dosage, duration, and timing), modality of exercise (cycling, rowing, and running), the intensity of exercise prescribed (high-intensity interval training and long slow distance training), duration of training study (acute exercise sessions and chronic exercise prescription), and training status of participants (trained and untrained). Variations within the outcome measures themselves (i.e., time trials and time to exhaustion) may contribute to conflicting findings (Laursen et al., 2007; Wylie et al., 2013). Therefore, continued investigation is needed to delineate the effects of nitrate supplementation on endurance performance.

### Effect of nitrate supplementation on resistance exercise performance

Although NO has largely been studied for its effect on endurance performance, researchers have begun to investigate its utility in resistance exercise performance. Tan et al. (2023b) recently performed a meta-analysis regarding the effects of nitrate supplementation on resistance exercise performance. The authors assessed the effect of nitrate supplementation on repetitions to failure, power output, and contraction velocity. Their analysis indicated statistically significant improvements in repetitions to failure but not in peak power output and peak velocity (Figure 6). Note that the description of the effects of nitrate supplementation on power output here refers specifically to power output during resistance training.

#### Repetitions to failure

Mosher and colleagues conducted a study examining the effect of nitrate supplementation on repetitions to failure in resistance exercise. In this study, 12 resistance-trained men completed two testing sessions where they performed three sets of bench presses at 60% one repetition maximum to failure. Either a nitrate supplement or a placebo was consumed before the commencement of each testing session in a randomized order. Participants’ rate of perceived exertion was recorded after each set, and blood lactate concentration was measured before and after the completion of each testing session. The results revealed a statistically significant increase in repetitions to failure and total weight lifted, while the rate of perceived exertion and blood lactate concentration remained unchanged (Mosher et al., 2016). Considering these results, one may speculate that nitrate supplementation may provide an advantage in resistance exercise performed at submaximal (moderate) intensities.

A more recent study by Ranchal-Sanchez et al. reports similar results. In this randomized, crossover investigation, 12 resistance-trained young adult men consumed either a nitrate supplement or a placebo before completing one set to failure at 60%, 70%, and 80% of their one repetition maximum in back squats and bench presses. Nitrate supplementation resulted in a statistically significant increase in repetitions to failure in back squats only at 60% and 70% one repetition maximum, although repetitions to failure at 80% one repetition maximum were unaffected. Additionally, bench press performance was unaltered following nitrate supplementation. Interestingly, although greater work was performed following nitrate supplementation (via increased repetitions to failure), both the rate of perceived exertion and post-exercise blood lactate concentration remained unchanged (Ranchal-Sanchez et al., 2020). Taken together, these data support the idea that nitrate supplementation may augment resistance training performance and, thus, provide an advantage in enhancing resistance exercise adaptation.

#### Power output

A study conducted by Rodriguez-Fernandez et al. observed an increase in mean power output and peak power output with nitrate supplementation. In this study, 18 recreationally active adult male candidates completed two testing sessions where they performed four sets of eight maximal repetitions, with each set adjusted to a different inertial load. Mean and peak power measurements were recorded during each repetition performed. Participants consumed either a nitrate supplement or a placebo 2.5 h before testing in a randomized, crossover design. A statistically significant increase in both mean and peak power output was observed following nitrate supplementation (Rodríguez-Fernández et al., 2021).

#### Contraction velocity

Lastly, recent research by Williams and colleagues determined a positive effect of nitrate supplementation on contraction velocity. This study utilized a randomized, crossover design to assess velocity in bench press performance in 11 trained young adult male candidates. After consuming a nitrate supplement or a placebo, participants completed two sets of two repetitions at 70% performed with maximal explosive intent. A linear position transducer was attached to the barbell during the bench press exercise to measure the velocity. The authors determined that nitrate supplementation provided a statistically significant increase in mean velocity but not in peak velocity (Williams et al., 2020).

Despite these promising reports, conflicting conclusions from other studies preclude any clear consensus regarding the true effect of nitrate supplementation on resistance exercise performance. For example, a 2022 investigation by Tan and colleagues does not agree with the findings that nitrate supplementation increases repetitions to failure in resistance exercise. Utilizing a similar study design to the aforementioned resistance exercise studies, Tan et al. recruited 14 healthy, male adults to complete two sets of two repetitions at 70% one repetition maximum, followed by one set of repetitions to failure at 60% one repetition maximum in both back squats and bench presses. In a randomized crossover design, participants completed the exercise testing protocol on two separate days after consuming either a nitrate supplement or a placebo. The results indicated a statistically significant increase in repetitions to failure in the bench press only, while repetitions to failure, power, and velocity were unaffected in back squat performance (Tan et al., 2022).

Considering the similarities between the study designs and yet the disagreement in the conclusions between these studies, it is obvious that further investigation into the efficacy of nitrate supplementation and exercise performance is very necessary. Even a cursory glance at the literature involving nitrate supplementation and exercise performance reveals several possible factors that may drastically alter the outcome. Two key factors often debated within the literature are training status and training type. The current thought suggests that nitrate supplementation affords the greatest advantage in untrained individuals, but it is an effect that is diminished as the individual becomes more trained (Van De Walle and Vukovich, 2018). Furthermore, researchers suspect not only a unique effect but also a specific mechanism of action when nitrate supplementation is utilized in different modes of training (endurance vs. resistance) (Jones, 2014a; McMahon et al., 2017; Tan, Pennell, et al., 2023). Other avenues of moderation include primary fiber type recruited during exercise (Jones et al., 2016), nitrate supplementation dosage (Coggan et al., 2018; Van De Walle and Vukovich, 2018), duration of supplementation (Van De Walle and Vukovich, 2018), and the age of the individuals (Stanaway et al., 2017). For a comprehensive review of this topic, see Silva et al. (2022) and Silva et al. (2022).

### Future directions

Although research on NO and nitrate supplementation has made significant progress during the past 3 decades, there are still many avenues to be explored and gaps to be filled. There are several opportunities to expand the experimental design of research investigating nitrate supplementation and exercise. For example, research has largely focused on acute nitrate supplementation and its effect on endurance training performance. To the best of our knowledge, no studies have currently investigated the effect of chronic nitrate supplementation lasting longer than 2 weeks. Furthermore, considering the significant role that mitochondrial function and biogenesis play in long-term aerobic adaptation and exercise performance and the involvement of NO in these pathways, our understanding of NO and the application of nitrate supplementation would greatly benefit from continued investigation within these topics. Studies have also neglected to investigate the utility of nitrate supplementation and subsequent mitochondrial adaptations in participants who engage in resistance exercise training.

Additionally, investigation into the effects of nitrate supplementation in different populations is warranted. Despite evidence that NO bioavailability significantly decreases with age, research has yet to investigate the effect of nitrate supplementation in older adults. NO production is known to decrease with aging primarily via decrements in the NOS-dependent pathway (Pourbagher-Shahri et al., 2021; Shannon et al., 2022; Stanaway et al., 2017). A 4-year investigation by Sverdlov and colleagues evaluating markers of NO generation and its effect on cardiovascular function in older adults demonstrated that aging negatively affected these parameters (Sverdlov et al., 2014). Although the exact causes of said decrements in NO production with aging have not been fully elucidated, evidence implicates decreased availability of cofactors necessary for NO production, such as tetrahydrobiopterin (BH4), decreased expression and activity of eNOS, decreased availability of L-arginine (via increased arginase activity), and excessive superoxide production as potential culprits (Cau et al., 2012; Siervo et al., 2018). Therefore, nitrate supplementation bolstering the contribution of the NOS-independent pathway is likely to provide particular benefits in the bioavailability and function of NO in older adult populations. Thus, future research is needed in each of these areas.

Finally, future research should explore and employ more advanced and accurate analytical techniques to assess nitrite, nitrate, nitric oxide, and NO-derived oxidants in human biological samples. In a comprehensive review of preprocessing and analysis of nitrite and nitrate, Liu H. et al. (2023) recommend the use of dispersive liquid–liquid microextraction (DLLME) in nitrite and nitrate assays for its rapidity, cost efficiency, low environmental impact, and minimal use of extraction solvents. Other techniques reviewed include spectroscopic methods (spectrofluorometry, colorimetry, chemiluminescence, and Raman spectroscopy), HPLC methods, HPLC–MS, ion chromatography, capillary electrophoresis, paper-based analytical devices, electrochemical sensors, and GC–MS (Liu G. et al., 2023). For a comprehensive review of measurement techniques of nitrite and nitrate, see Liu H. et al. (2023).

### Limitations

Although this review has carefully summarized the literature on NO production, its function in vasculature, skeletal muscle, mitochondria, and redox state, and the effects of nitrate supplementation in endurance and resistance exercise, it is important to note that there are some limitations that have not been addressed. For example, research indicates that there is a dose-response relationship within nitrate supplementation that impacts its effects (Silva et al., 2022; Wylie et al., 2013). Research has yet to explore the effects of chronic daily nitrate supplementation lasting longer than 14 days. As apparent from differing results across numerous studies, it appears that there may be an interaction between the dose, timing, and duration of nitrate supplementation and other variables such as training status, sex, age, and mode of exercise (Anderson et al., 2022; Rothschild and Bishop, 2020; San Juan et al., 2020; Tan et al., 2023a; Tan et al., 2024).

## Conclusion

In summary, NO is a ubiquitous molecule continuously produced in the human body, serving many essential functions. It is produced either with the aid of NOS enzymes in the NOS-dependent pathway or formed from dietary nitrate in the NOS-independent pathway. Although some aspects of its involvement are unclear, NO is known to aid in vascular, skeletal muscle, and mitochondrial function as well as in maintaining the redox balance. Nitrate supplementation is believed to enhance exercise performance via its involvement at these locations within the whole-body system, tissues, cells, and individual molecular reactions. Ongoing research is aimed at elucidating the specific effects of nitrate supplementation on endurance and resistance exercise performance.
